# Real-world treatment patterns and outcomes of patients with LR-MDS in Japan: an electronic health record database study

**DOI:** 10.1007/s12185-025-04077-y

**Published:** 2025-10-02

**Authors:** Shuji Uno, Mayuko Nakakoji, Shuichi Midorikawa, Takuya Kitano, Ryo Tajiri, Jin Hayakawa, Kenichi Minehata, Takahiro Suzuki

**Affiliations:** 1 Bristol Myers Squibb, K.K., Otemachi One Tower, 1-2-1 Otemachi, Chiyoda-ku, Tokyo, 100-0004 Japan; 2 Mebix, Inc., Tokyo, Japan; 3 Kitasato University School of Medicine, Sagamihara, Japan

**Keywords:** Healthcare resource utilization, Luspatercept, Myelodysplastic syndromes, Outcomes, Treatment patterns

## Abstract

Real-world treatment patterns and clinical outcomes for patients with lower-risk myelodysplastic syndromes (LR-MDS) in Japan are insufficiently characterized. This study assessed treatment patterns, clinical outcomes, costs, and healthcare resource utilization of patients with LR-MDS in Japan using the AsMedix electronic health record database. The study included 177 patients aged ≥ 18 years with LR-MDS, identified between April 1, 2017, and March 31, 2022. Of these, 79 were transfusion dependent (TD) and 98 were non-transfusion dependent (NTD). Treatment patterns were diverse, and 57 (32.2%) patients received hypomethylating agents (combination or monotherapy) as first-line treatment. Median (95% confidence interval) overall survival was 32.6 months (17.6–not evaluable) among TD patients who achieved red blood cell transfusion independence (RBC-TI) ≥ 8 weeks during weeks 1–24, compared with 22.3 months (10.1–not evaluable) among those who did not (*P* = 0.17). Among NTD patients, maintaining NTD status was also associated with longer median overall survival. Furthermore, patients who achieved or maintained RBC-TI incurred roughly half the monthly medical costs per individual compared with those who did not, highlighting the clinical and economic importance of reducing RBC transfusion requirements. Further research is needed to understand the impacts of treatment on patients with LR-MDS-related anemia in Japan.

**Supplementary Information:**

The online version contains supplementary material available at 10.1007/s12185-025-04077-y.

## Introduction

Myelodysplastic syndromes (MDS), also known as myelodysplastic neoplasms, are a heterogeneous group of hematologic malignancies that are characterized by ineffective hematopoiesis, morphologic dysplasia, and cytopenias [1–4].

Most patients with MDS develop symptomatic anemia [3, 5, 6], and a key goal of treatment is addressing this anemia [3, 7]. As a result of anemia, patients with lower risk MDS (LR-MDS) often require red blood cell (RBC) transfusions [3]. RBC transfusion dependence is associated with an increased risk of comorbidities, hospitalization, and death [4, 5, 8]. RBC transfusions also negatively affect the clinical, humanistic, and economic outcomes of patients with LR-MDS, as well as their quality of life [2, 4, 5, 8]. While Japan’s Ministry of Health, Labor and Welfare (MHLW) Guidelines for the Use of Blood Products [9] highlight the importance of treating patients early before they become transfusion dependent (TD) to reduce the number of transfusions required, it is unclear to what extent transfusions can be avoided in real-world practice or what effect this would have on overall survival outcomes and medical costs in a Japanese healthcare setting.

Treatment recommendations, including RBC transfusion practices, differ between countries, potentially leading to differences in patient outcomes. The Japanese Society of Hematology guidelines for the treatment of symptomatic LR-MDS recommend the use of treatments including cytokine therapy, such as erythropoiesis-stimulating agents (ESAs; e.g., erythropoietin or darbepoetin alpha) for patients with low serum EPO levels, granulocyte colony-stimulating factor for limited use (e.g., during infection), and lenalidomide for patients with MDS del(5q). Azacitidine is not recommended as a first-line treatment to prolong survival [10]. In 2024, a new treatment option, luspatercept, an erythroid maturation agent, was approved in Japan for the treatment of patients with anemia associated with MDS [11, 12] and is now included in the Japanese Society of Hematology’s guideline recommendations [10]. For those patients with low-risk disease without clinical symptoms, observation is recommended [10].

Analyzing electronic health record (EHR) databases or claims databases is considered useful to understand the impacts and value of newly launched drugs for medical care. It is necessary to consider treatment goals and recommended treatments by lower-risk versus higher-risk MDS because the treatment goals and recommended treatments differ by risk status [10]. However, claims databases in Japan do not contain information on MDS risk status [13]. Therefore, an approach was taken to utilize an EHR/Claims Integrated Database provided by AsMedix, which provides information on MDS risk status from medical records.

In this study, we aimed to assess the real-world treatment patterns, clinical outcomes, costs, and healthcare resource utilization of patients with LR-MDS in Japan using an EHR database.

## Materials and methods

### Data source

This retrospective observational study used deidentified EHR and health claims data from a database provided by AsMedix Co., Ltd (Tokyo, Japan). The database consists of records from patients treated in 8 hospitals across Japan (treating a total of approximately 1,445,354 inpatients and 1,996,712 outpatients annually), including private and public clinics and large medical centers (300–1000 beds), all with hematologists present, covering geographic regions from Hokkaido (North Japan) to Okinawa (South Japan). Data collected included demographics, clinical characteristics, and medical and pharmacy claims. Data from participating institutions were compiled by AsMedix into a single dataset. During this process, data were standardized, cleaned, and integrated, thereby allowing data to be linked for each case. Test data received from different hospitals were converted to standard test value units.

### Study population

Eligible patients were aged 18 years or older at the index date (defined as the date of the patient’s first LR-MDS-related treatment after diagnosis); were diagnosed with MDS (defined as International Classification of Diseases, 10th revision, code D46) between May 1, 2017, and January 31, 2022; had their diagnosis confirmed as lower risk (defined as either Low [0 points] or Intermediate-1 [> 0 to 1 point] per International Prognostic Scoring System (IPSS); Very low [≤ 1.5 points], Low [> 1.5 to 3 points], or Intermediate [> 3 to 4.5 points] per Revised International Prognostic Scoring System (IPSS-R) [14, 15]; or EHR mention of Very Low, Low, Intermediate, Intermediate-1, or LR-MDS); and had a bone marrow procedure (code, D404) performed from 30 days before to 30 days after the date of diagnosis. Exclusion criteria included age < 18 years at the index date and having an observation period < 30 days since the initial diagnosis of MDS. Patient IDs that were listed as lower risk by the database provider (AsMedix) were extracted along with the risk information; the risk information of each case was then reviewed.

### Study design

**Fig. 1:**
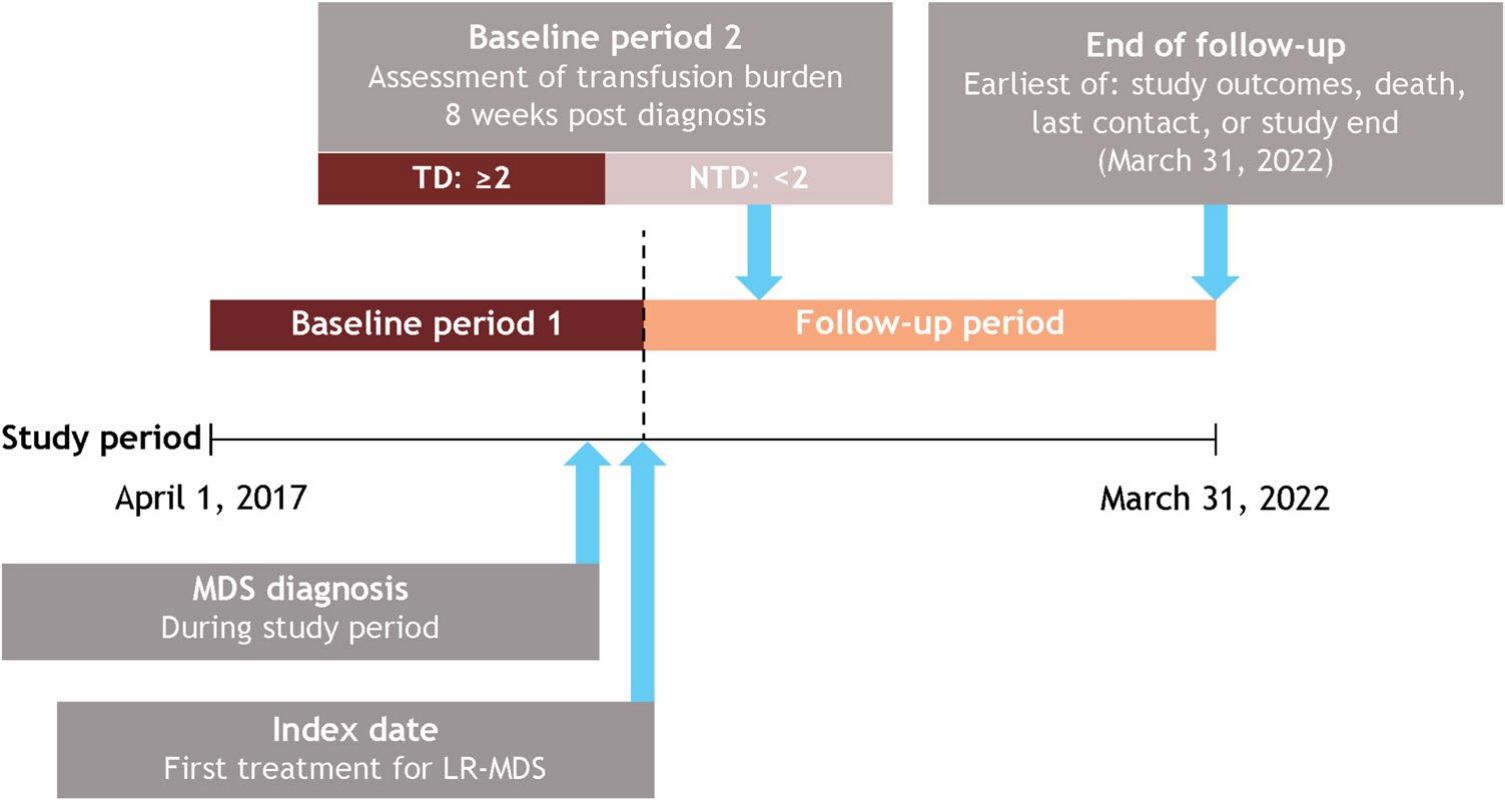
Study design. *LR-MDS* lower-risk myelodysplastic syndromes, *MDS* myelodysplastic syndromes, *NTD* non-transfusion dependent, *TD* transfusion dependent. Adapted from Uno S, et al. Poster presentation at the 86th Annual Meeting of the Japanese Society of Hematology (JSH). Kyoto, Japan; October 11–13, 2024. Poster P-1–5-1 [16].

The study period was April 1, 2017, to March 31, 2022, and included 2 baseline periods and a follow-up period (Fig. 1). Baseline period 1 was defined as the period 30 days before the diagnosis date to either 30 days after the diagnosis date or one day before the index date, whichever came first. If multiple data were available, the data closest to the diagnosis date were used to assess demographic and clinical characteristics. Baseline period 2 was defined as the 8 weeks following the initial diagnosis of MDS, during which baseline transfusion status was assessed. The follow-up period was defined as the time from the index date (date of the patient’s first LR-MDS-related treatment after diagnosis) until whichever of the following occurred first: outcomes under study, death, date of last contact, or end of study (March 31, 2022).

### Outcomes

Patients were stratified by baseline RBC transfusion burden in the baseline period 2 (8 weeks following the initial diagnosis of MDS) based on clinical expert opinion. Patients who required < 2 RBC units during these 8 weeks, including patients who did not receive any RBC transfusions in the 8 weeks following diagnosis, were classified as non-transfusion dependent (NTD), and patients who required ≥ 2 RBC units during these 8 weeks were classified as TD based on previous studies and expert opinion.

Treatment patterns, specifically first-line treatment choice, were analyzed by transfusion status (TD vs NTD). "Watchful waiting" during first-line treatment among NTD patients was defined as having no record of MDS-related treatment during the study period. Mean hemoglobin changes from baseline at 24 and 48 weeks were also analyzed, and a 14/3-day rule was applied in which only hemoglobin values measured ≥ 14 days after a transfusion were used, unless there was another transfusion within the 3 days after hemoglobin assessment (in which case the second hemoglobin value was used); if hemoglobin records were missing after applying the 14/3-day rule, a 7/3-day rule was applied instead. Achievement of RBC transfusion independence (RBC-TI; defined as a requirement of < 2 RBC units for ≥ 8, ≥ 12, and ≥ 24 weeks) was evaluated among TD patients. Maintenance of RBC-TI status for ≥ 24, ≥ 48, and ≥ 72 weeks was evaluated among NTD patients. Overall survival was analyzed for all patients.

Healthcare resource utilization outcomes included the number of outpatient and inpatient visits (including emergency room and intensive care unit visits), number of RBC transfusions received, total number of RBC units transfused, and total direct adjusted medical costs.

Costs are reported in Japanese yen (JPY) and US dollars (USD). JPY were converted into USD using the exchange rate during the first month of the corresponding calendar year. In addition, as the costs occurred across different calendar years, cost data are presented after being adjusted by the average Consumer Price Index for each corresponding calendar year. The datasets for medical costs provided for this study were separate inpatient and outpatient datasets based on the Diagnosis Procedure Combination system.

### Statistical analysis

Baseline demographic and clinical characteristics were summarized using descriptive statistics; categorical variables were reported as frequency, whereas percentages and continuous variables were reported as mean and standard deviation or median and range, interquartile range (IQR), or 95% confidence interval (CI). Overall survival was assessed using Kaplan–Meier curves. Healthcare resource utilization data were reported as mean values per person per month (PPPM) and 95% CI. All analyses were performed using SAS v9.4 (SAS Institute, Cary, NC, USA) and R Statistical Software (v4.2.2; R Core Team 2024).

## Results

### Patient population and baseline characteristics

**Fig. 2:**
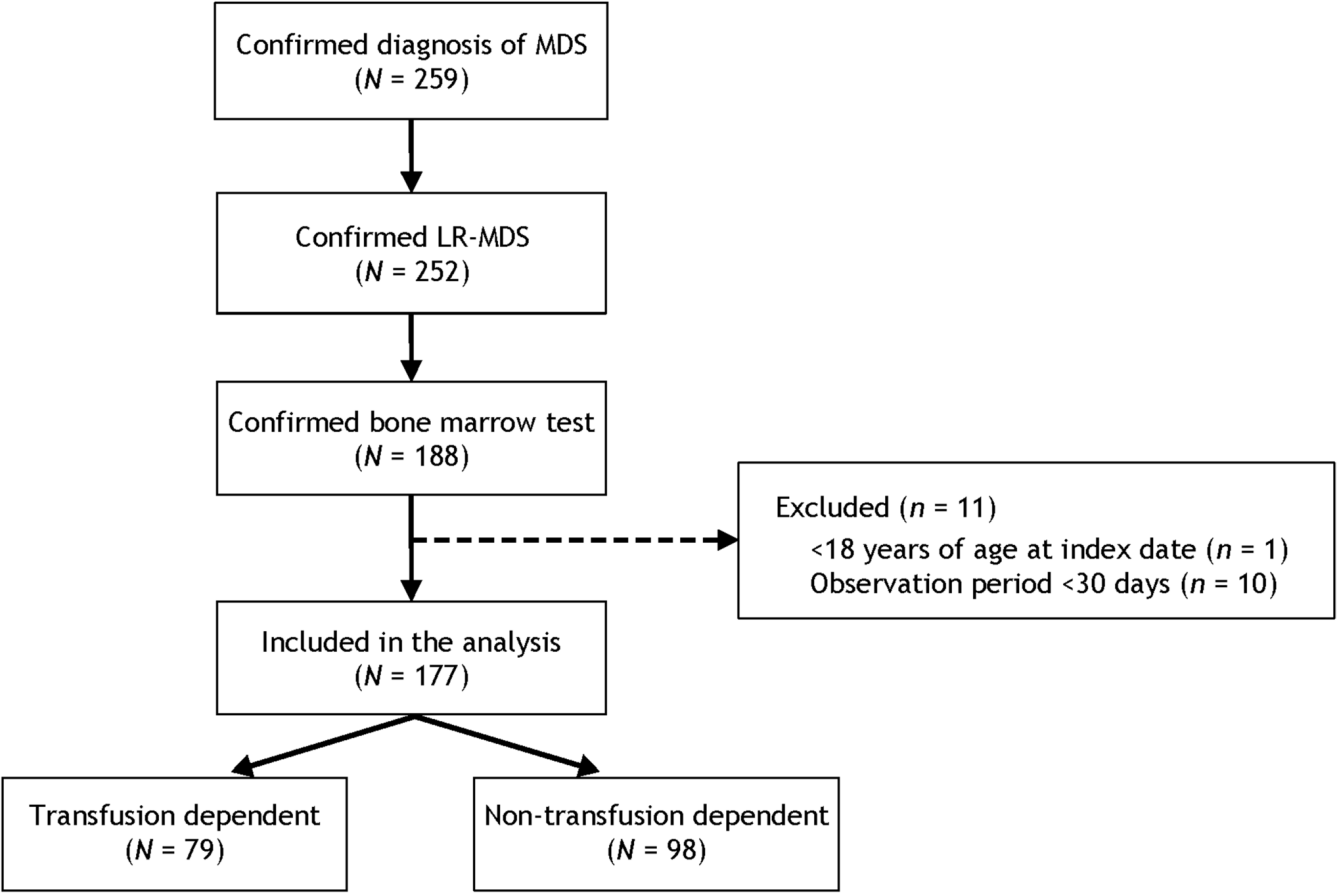
Patient disposition. *LR-MDS* lower-risk myelodysplastic syndromes, *MDS* myelodysplastic syndromes. Adapted from Uno S, et al. Poster presentation at the 86th Annual Meeting of the Japanese Society of Hematology (JSH). Kyoto, Japan; October 11–13, 2024. Poster P-1–5-1 [16].

**Fig. 3:**
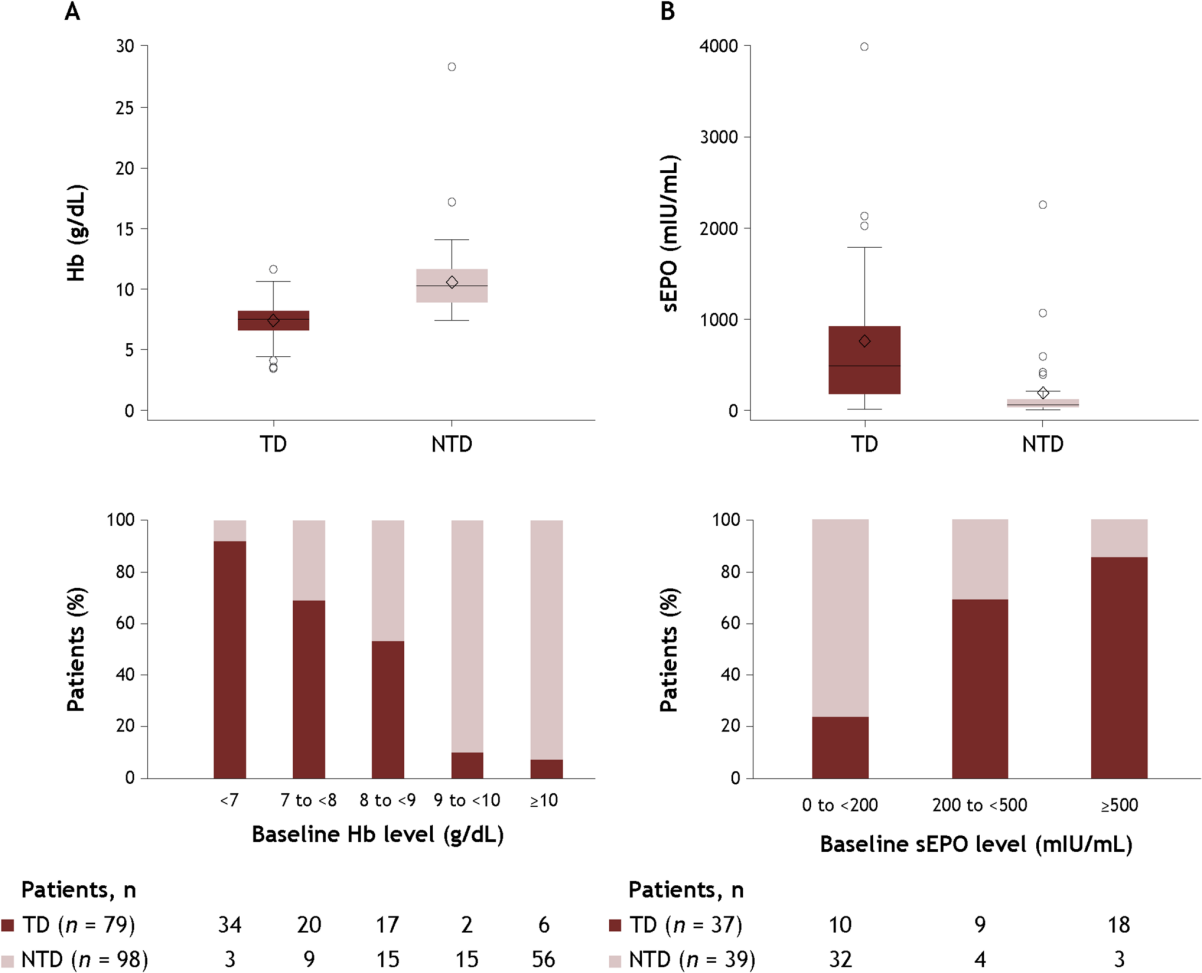
Distribution of baseline hemoglobin (**A**) and serum erythropoietin (**B**) levels by baseline transfusion status. *Hb* hemoglobin, *NTD* non-transfusion dependent, *sEPO* serum erythropoietin, *TD* transfusion dependent. Adapted from Uno S, et al. Poster presentation at the 86th Annual Meeting of the Japanese Society of Hematology (JSH). Kyoto, Japan; October 11–13, 2024. Poster P-1–5-1 [16].

A total of 177 patients with LR-MDS were included in the analysis, including 79 TD patients and 98 NTD patients (Fig. 2); all 98 NTD patients had no history of RBC transfusion during baseline period 2 when the baseline transfusion burden was evaluated. Patient baseline characteristics are shown in Table 1, and the IPSS and IPSS-R classifications are shown in Supplementary Tables 1 and 2. TD patients had a lower median hemoglobin level (7.3 g/dL vs 10.3 g/dL) and a higher serum erythropoietin level (482.0 mIU/mL [*n* = 37] vs 59.8 mIU/mL [*n* = 39]) than NTD patients, respectively. The distribution of baseline hemoglobin and serum erythropoietin levels by baseline transfusion dependency is shown in Fig. 3. The median duration of observation was 491 days overall, 434 days in TD patients, and 671 days in NTD patients.

**Table 1:** Baseline demographic and clinical characteristics

	All patients (*N* = 177)	TD patients (*n* = 79)	NTD patients (*n* = 98)
Age, median (range), years	76.0 (23–88)	74.0 (23–88)	76.5 (38–88)
Male, *n* (%)	113 (63.8)	49 (62.0)	64 (65.3)
ECOG PS score, *n* (%)			
0	2 (1.1)	2 (2.5)	0
1	7 (4.0)	5 (6.3)	2 (2.0)
2	0	0	0
3	0	0	0
4	1 (0.6)	1 (1.3)	0
CCI score, mean (SD)	1.6 (2.1)	1.6 (2.3)	1.6 (2.1)
CCI score, *n* (%)			
0–1	103 (58.2)	47 (59.5)	56 (57.1)
2	30 (16.9)	15 (19.0)	15 (15.3)
3	16 (9.0)	8 (10.1)	8 (8.2)
≥ 4	28 (15.8)	9 (11.4)	19 (19.4)
Hb, median (IQR), g/dL	8.6 (7.4–10.6)	7.3 (6.3–8.2)	10.3 (8.7–11.6)
sEPO, median (IQR), mIU/mL	***n* = 76** 118.0 (49.1–580.5)	***n* = 37** 482.0 (175.0–911.0)	***n* = 39** 59.8 (36.1–119.0)
Serum ferritin, median (IQR), ng/mL	***n* = 139** 283.0 (142.1–493.3)	***n* = 64** 328.1 (212.7–583.8)	***n* = 75** 185.0 (87.4–387.0)
Neutrophil count, median (IQR), per μL	***n* = 88** 2314.0 (733.5–5450.0)	***n* = 35** 2360.0 (663.0–5500.0)	***n* = 53** 2268.0 (924.0–5400.0)
Platelet count, median (IQR), × 10^4^/μL	9.9 (4.9–16.8)	8.4 (2.8–18.2)	10.5 (6.4–16.5)
Creatinine, median (IQR), mg/dL	***n* = 176** 0.79 (0.65–1.01)	***n* = 79** 0.85 (0.66–1.05)	***n* = 97** 0.76 (0.65–1.0)
eGFR, median (IQR), mL/min/1.73 m^2^	***n* = 173** 65.3 (49.5–82.1)	***n* = 77** 60.7 (47.3–74.0)	***n* = 96** 69.3 (53.0–83.0)
BNP, median (IQR), pg/mL	***n* = 69** 78.1 (42.9–161.6)	***n* = 41** 92.7 (48.7–166.0)	***n* = 28** 62.8 (34.0–140.7)
NT-proBNP, median (IQR), pg/mL	***n* = 18** 328.5 (123.0–819.0)	***n* = 14** 524.5 (127.0–1258.0)	***n* = 4** 165.5 (62.0–283.0)

The bold font indicates differing n values for the categories

Adapted from Uno S, et al. Poster presentation at the 86th Annual Meeting of the Japanese Society of Hematology (JSH). Kyoto, Japan; October 11–13, 2024. Poster P-1–5-1 [16]

The observation period spanned 30 days before the date of the first MDS diagnosis, and data closest to the MDS diagnosis date were used. If data were not available during this period, data closest to the diagnosis date in the 30 days after diagnosis were used

*BNP* brain natriuretic peptide, *CCI* Charlson Comorbidity Index, *ECOG PS* Eastern Cooperative Oncology Group performance status, *eGFR* estimated glomerular filtration rate, *Hb* hemoglobin, *IQR* interquartile range, *MDS* myelodysplastic syndromes, *NTD* non-transfusion dependent, *NT-proBNP* N-terminal prohormone of BNP, *SD* standard deviation, *sEPO* serum erythropoietin, *TD* transfusion dependent

Patients who were treated with hypomethylating agents (HMAs) as their first-line therapy had a median baseline hemoglobin level of 7.8 g/dL (IQR 6.6–9.9), median baseline neutrophil count of 1615.0/μL (IQR 800.0–5475.0), and median baseline platelet count of 7.6 × 10^4^/μL (IQR 2.8–12.4); in all cases, these values were lower among patients treated with HMAs than among patients treated with ESAs as their first-line therapy (Supplementary Table 3).

### Treatment patterns

**Fig. 4:**
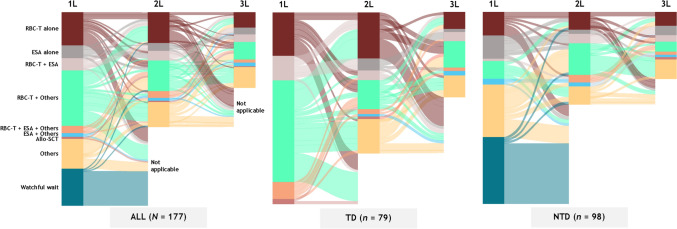
Treatment patterns*.* “Others” includes immunosuppressive agents, anabolic steroids, hypomethylating agents, immunomodulatory agents, granulocyte colony-stimulating factors, and vitamins. *1L* first line*, 2L* second line*, 3L* third line*, Allo-SCT* allogenic stem cell transplant, *ESA* erythropoiesis-stimulating agent, *RBC-T* red blood cell transfusion

The median time from diagnosis to the first LR-MDS-related treatment (index date) was 0.16 months (IQR 0–1.15) overall, 0.10 months (IQR 0–0.33) for TD patients, and 0.61 months (IQR 0–3.75) for NTD patients. More than half of the patients (99 of 177 patients, 55.9%) received RBC transfusions as first-line treatment. Fifty-seven (32.2%) patients received HMAs, and 33 (18.6%) patients received ESAs (Table 2). Of the 98 NTD patients, 34 (34.7%) patients without first-line anemia treatment (“watchful waiting”) were followed (Fig. 4). For TD patients, lines of therapy 2 and 3 primarily consisted of RBC transfusions alone or in combination with another treatment.

**Table 2:** First-line treatment patterns overall and by baseline transfusion status

First-line treatment, *n* (%)	All patients (*N* = 177)	TD patients (*n* = 79)	NTD patients (*n* = 98)
ESA alone	12 (6.8)	0	12 (12.2)
Other treatment^a^	27 (15.3)	0	27 (27.6)
ISA	2 (1.1)	0	2 (2.0)
AS	7 (4.0)	0	7 (7.1)
HMA	17 (9.6)	0	17 (17.3)
IMiD agent	1 (0.6)	0	1 (1.0)
G-CSF	1 (0.6)	0	1 (1.0)
Vitamin	5 (2.8)	0	5 (5.1)
ESA + other	3 (1.7)	0	3 (3.1)
ESA + ISA	0	0	0
ESA + AS	1 (0.6)	0	1 (1.0)
ESA + HMA	1 (0.6)	0	1 (1.0)
ESA + IMiD agent	0	0	0
ESA + G-CSF	0	0	0
ESA + vitamin	1 (0.6)	0	1 (1.0)
RBC transfusion alone	30 (16.9)	18 (22.8)	12 (12.2)
RBC transfusion + ESA	11 (6.2)	10 (12.7)	1 (1.0)
RBC transfusion + other^a^	51 (28.8)	42 (53.2)	9 (9.2)
RBC transfusion + ISA	12 (6.8)	10 (12.7)	2 (2.0)
RBC transfusion + AS	8 (4.5)	6 (7.6)	2 (2.0)
RBC transfusion + HMA	33 (18.6)	29 (36.7)	4 (4.1)
RBC transfusion + IMiD agent	3 (1.7)	3 (3.8)	0
RBC transfusion + G-CSF	17 (9.6)	13 (16.5)	4 (4.1)
RBC transfusion + vitamin	4 (2.3)	3 (3.8)	1 (1.0)
RBC transfusion + ESA + other^a^	7 (4.0)	7 (8.9)	0
RBC transfusion + ESA + ISA	2 (1.1)	2 (2.5)	0
RBC transfusion + ESA + AS	3 (1.7)	3 (3.8)	0
RBC transfusion + ESA + HMA	6 (3.4)	6 (7.6)	0
RBC transfusion + ESA + IMiD agent	0	0	0
RBC transfusion + ESA + G-CSF	1 (0.6)	1 (1.3)	0
RBC transfusion + ESA + vitamin	3 (1.7)	3 (3.8)	0
ASCT	2 (1.1)	2 (2.5)	0
Watchful waiting	34 (19.2)	0	34 (34.7)

Adapted from Uno S, et al. Poster presentation at the 86th Annual Meeting of the Japanese

Society of Hematology (JSH). Kyoto, Japan; October 11–13, 2024. Poster P-1–5-1 [16]

*AS* anabolic steroid, *ASCT* allogeneic stem cell transplant, *ESA* erythropoiesis-stimulating agent, *G-CSF* granulocyte colony-stimulating factor, *HMA* hypomethylating agent, *IMiD* immunomodulatory drug*, ISA* immunosuppressive agent, *NTD* non-transfusion dependent, *RBC* red blood cell, *TD* transfusion dependent

^a^Patients could receive more than one “Other” treatment

### Clinical outcomes

Rates of RBC-TI achievement among TD patients are shown in Table 3. In weeks 1–24, 35 of 68 evaluable patients (51.5%) and 31 of 71 evaluable patients (43.7%) achieved RBC-TI ≥ 8 weeks and ≥ 12 weeks, respectively; no patient achieved RBC-TI ≥ 24 weeks. In weeks 1–48, 40 of 51 evaluable patients (78.4%), 36 of 50 evaluable patients (72.0%), and 25 of 57 evaluable patients (43.9%) achieved RBC-TI ≥ 8 weeks, ≥ 12 weeks, and ≥ 24 weeks, respectively.

**Table 3:** Achievement of RBC-TI among TD patients across all lines of therapy

TD patients, *n* (%)	Weeks 1–24 (*n* = 79)	Weeks 1–48 (*n* = 79)
RBC-TI for 8 weeks (56 consecutive days)		
Evaluable patients	68 (86.1)	51 (64.6)
Responder	35 (51.5)	40 (78.4)
Non-responder	33 (48.5)	11 (21.6)
Non-evaluable patients	11 (13.9)	28 (35.4)
RBC-TI for 12 weeks (84 consecutive days)		
Evaluable patients	71 (89.9)	50 (63.3)
Responder	31 (43.7)	36 (72.0)
Non-responder	40 (56.3)	14 (28.0)
Non-evaluable patients	8 (10.1)	29 (36.7)
RBC-TI for 24 weeks (168 consecutive days)		
Evaluable patients	79 (100)	57 (72.2)
Responder	0	25 (43.9)
Non-responder	79 (100)	32 (56.1)
Non-evaluable patients	0	22 (27.8)

Adapted from Uno S, et al. Poster presentation at the 86th Annual Meeting of the Japanese Society of Hematology (JSH). Kyoto, Japan; October 11–13, 2024. Poster P-1–5-1 [16]

Patients were considered responders if they did not receive RBC transfusions during the relevant period (weeks 1–8, weeks 1–12, and weeks 1–24) and were considered non-responders if they remained TD

*RBC* red blood cell, *RBC-TI* RBC transfusion independence, *TD* transfusion dependent

Rates of maintenance of NTD status and conversion to TD status among NTD patients are shown in Table 4. Of 98 NTD patients, 35 of 83 evaluable patients (42.2%), 41 of 78 evaluable patients (52.6%), and 42 of 70 evaluable patients (60.0%) received RBC transfusions in weeks 1–24, weeks 1–48, and weeks 1–72, respectively.

**Table 4:** Maintenance of NTD status and conversion to TD status among NTD patients across all lines of therapy

NTD patients, *n* (%)	Weeks 1–24 (*n* = 98)	Weeks 1–48 (*n* = 98)	Weeks 1–72 (*n* = 98)
Evaluable patients	83 (84.7)	78 (79.6)	70 (71.4)
No RBC transfusion	48 (57.8)	37 (47.4)	28 (40.0)
Started RBC transfusion	35 (42.2)	41 (52.6)	42 (60.0)
Non-evaluable patients	15 (15.3)	20 (20.4)	28 (28.6)

Patients were considered to maintain NTD if they did not receive RBC transfusions during the relevant period (weeks 1–24, weeks 1–48, and weeks 1–72)

*NTD* non-transfusion dependent, *RBC* red blood cell, *RBC-TI* RBC transfusion independence, *TD* transfusion dependent

To better understand the progression of anemia among NTD patients requiring treatment other than RBC transfusions, patients who were not subject to “watchful waiting” but instead started their first-line treatment without RBC transfusions (*n* = 42) were also examined. Among the 42 eligible patients, 12 of 35 patients (34.3%), 18 of 35 patients (51.4%), and 19 of 32 patients (59.4%) had initiated RBC transfusions in weeks 1–24, weeks 1–48, and weeks 1–72, respectively (Supplementary Table 4).

Mean hemoglobin change from baseline for TD and NTD patients is shown in Supplementary Table 5. Across all lines of therapy, 22.8% (week 24) and 24.1% (week 48) of TD patients showed an increase in hemoglobin levels of ≥ 1.5 g/dL compared with 5.1% (week 24) and 7.1% (week 48) of NTD patients.

### Survival outcomes

The median overall duration of follow-up was 491 days (range 37–1771). Median overall survival was not reached among all patients (95% CI 27.5–not evaluable). Median overall survival was 22.3 months (95% CI 18.8–not evaluable) among TD patients and was not reached (95% CI 34.1–not evaluable) among NTD patients (*P* = 0.0023; Supplementary Fig. 1).

**Fig. 5:**
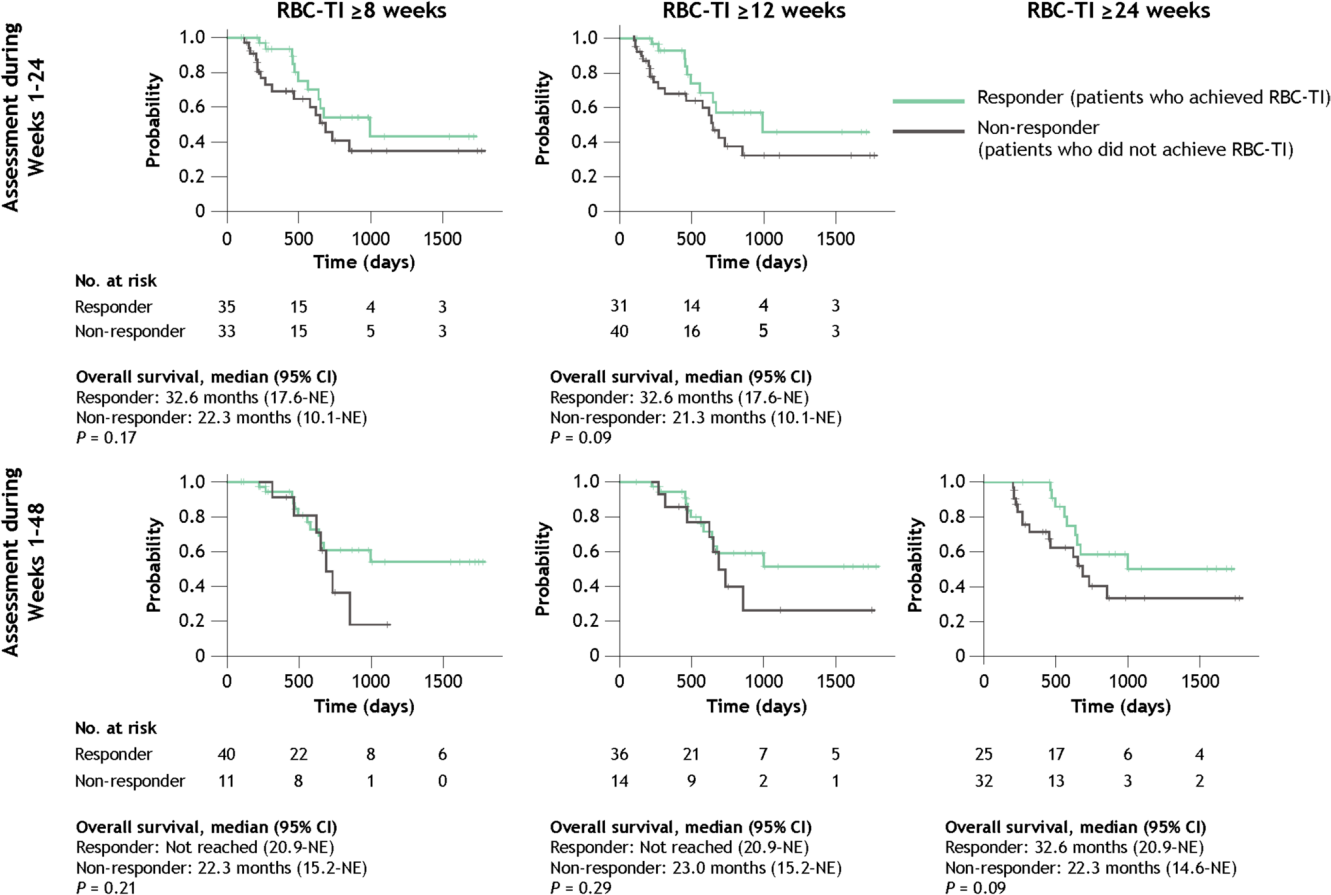
Overall survival in TD patients who achieved RBC-TI and those who did not. *CI* confidence interval, *NE* not evaluable, *RBC-TI* red blood cell transfusion independence, *TD* transfusion dependent. Adapted from Uno S, et al. Poster presentation at the 86th Annual Meeting of the Japanese Society of Hematology (JSH). Kyoto, Japan; October 11–13, 2024. Poster P-1–5-1 [16].

**Fig. 6:**
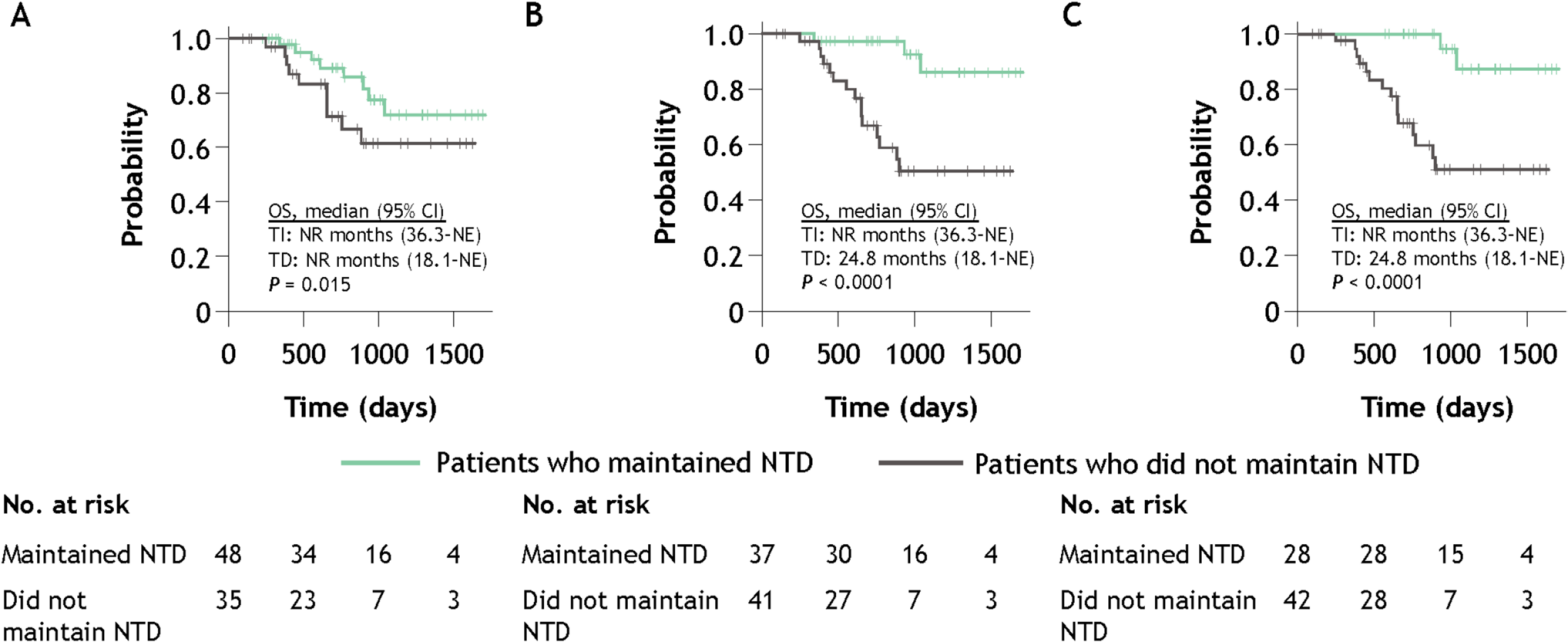
Overall survival among NTD patients who maintained NTD status and those who did not from weeks 1–24 (**A**), weeks 1–48 (**B**), and weeks 1–72 (**C**). *CI* confidence interval, *NR* not reached, *NTD* non-transfusion dependent, *OS* overall survival, *TD* transfusion dependent, *TI* transfusion independent. Adapted from Uno S, et al. Poster presentation at the 86th Annual Meeting of the Japanese Society of Hematology (JSH). Kyoto, Japan; October 11–13, 2024. Poster P-1–5-1 [16].

Median overall survival was 32.6 months (95% CI 17.6–not evaluable) among TD patients who achieved RBC-TI ≥ 8 weeks during weeks 1–24 and was 22.3 months (95% CI 10.1–not evaluable) among those who did not (*P* = 0.17; Fig. 5). Median overall survival was not reached among NTD patients who maintained NTD status during weeks 1–24 (95% CI 36.3–not evaluable) and among those who did not maintain NTD status during weeks 1–24 (95% CI 18.1–not evaluable) (*P* = 0.015; Fig. 6).

### Healthcare resource utilization

Total direct adjusted medical costs PPPM were JPY 498,230.73 (95% CI 498,206.14–498,255.31; total USD 4612.99) for all patients, JPY 728,799.41 (95% CI 728,753.24–728,845.58; total USD 6746.71) for TD patients, and JPY 334,797.30 (95% CI 334,770.95–334,823.65; total USD 3100.56) for NTD patients. TD patients who were categorized as responders (i.e., achieved RBC-TI for ≥ 8 weeks during weeks 1–24) had lower medical costs compared with non-responders: the average direct adjusted medical costs PPPM were JPY 600,995.97 (95% CI 600,946.65–601,045.28; total USD 5559.61) for TD responders and JPY 1,062,169.56 (95% CI 1,062,063.68–1,062,275.45; total USD 9843.20) for TD non-responders. Among NTD patients, the average direct adjusted medical costs PPPM were JPY 254,156.04 (95% CI 254,127.95–254,184.14; total USD 2354.32) for those who maintained non-transfusion dependence for ≥ 24 weeks and JPY 496,721.50 (95% CI 496,665.84–496,777.16; total USD 4598.97) for those who did not (Supplementary Table 6).

Patients had a total of 5718 outpatient visits overall (mean 1.81 visits PPPM, 95% CI 1.76–1.85). Among TD patients, there were a total of 2307 outpatient visits (mean 1.76 visits PPPM, 95% CI 1.68–1.83), compared with a total of 3411 outpatient visits among NTD patients (mean 1.84 visits PPPM, 95% CI 1.78–1.90). Patients had 520 inpatient visits overall (mean 0.16 visits PPPM, 95% CI 0.15–0.18). TD patients had a total of 278 inpatient visits (mean 0.21 visits PPPM, 95% CI 0.19–0.24), compared with a total of 242 inpatient visits among NTD patients (mean 0.13 visits PPPM, 95% CI 0.11–0.15).

Patients received a total of 2419 RBC transfusions overall (mean 0.76 RBC transfusions PPPM, 95% CI 0.73–0.79). TD patients received a total of 1760 RBC transfusions (mean 1.34 RBC transfusions PPPM, 95% CI 1.28–1.40) compared with a total of 659 RBC transfusions among NTD patients (mean 0.36 RBC transfusions PPPM, 95% CI 0.33–0.38). The total number of RBC units transfused was 5118 (mean 1.62 RBC units PPPM, 95% CI 1.57–1.66) overall, 3738 (mean 2.85 RBC units PPPM, 95% CI 2.76–2.94) for TD patients, and 1380 (mean 0.74 RBC units PPPM, 95% CI 0.71–0.78) for NTD patients.

## Discussion

To our knowledge, this is the first study to analyze real-world treatment patterns and clinical outcomes of patients with LR-MDS in Japan using an EHR database.

Due to the nature of data originating from an EHR database, the study population was examined to consider its relevance in the context of previously reported studies. Median age at baseline (76 years old) in this study was comparable to that of previous studies of patients with MDS in Japan [13, 17–19], suggesting that the data analyzed in this study are reflective of patient characteristics in Japan.

Baseline hemoglobin and serum erythropoietin levels were also similar to those observed in previous studies [20, 21]. Although a correlation between hemoglobin and serum erythropoietin levels was previously reported in patients with LR-MDS [21], no such correlation was observed in this study population, even when patients with impaired renal function were excluded (data not shown).

Cardiovascular disease is the second most common cause of death in the LR-MDS population, and an N-terminal prohormone brain natriuretic peptide (NT-proBNP) level ≥ 486 pg/mL has been reported as an independent predictor of poor cardiovascular prognosis [22]. In Japan, the standard value for NT-proBNP is considered to be below 125 pg/mL [23]. In this study, however, the median NT-proBNP values were 328.5 pg/mL overall, 165.5 pg/mL in NTD patients, and 524.5 pg/mL in TD patients. The elevated NT-proBNP levels seen in these TD patients are consistent with other published studies; in a study of cardiovascular disease among TD patients with LR-MDS, half of the 31 patients had high NT-proBNP levels (> 400 pg/mL) at first evaluation [22], placing them at an increased risk for adverse cardiovascular outcomes. These observations suggest that there is an unmet need for avoiding RBC transfusions among patients with LR-MDS, especially in TD patients, due to strain on the heart.

A relatively large proportion of TD patients received RBC transfusions either alone or in combination with HMAs or ESAs as first-line treatment, and this pattern continued into second- and third-line treatment. Although ESA therapy (darbepoetin alpha) is covered by health insurance in Japan, in this study, it was used by fewer than a third of patients. Perhaps one reason behind this could be that ESAs are known to be less effective when serum erythropoietin levels are high [24], such as those over 200 or 500 mIU/mL; TD patients in this study population had high serum erythropoietin levels at the time of diagnosis.

On the other hand, although HMAs are not among the treatments recommended for use in patients with LR-MDS [10], they were found to be frequently used in this study (received by 32.2% of patients). This may be attributable to the subset of patients who are not eligible to receive ESAs due to the levels of hemoglobin and serum erythropoietin [25]. They may also have been used in patients with multiple lineage cytopenias. Hemoglobin, platelet counts, and neutrophil counts in patients receiving HMAs were lower than those in patients receiving ESAs.

The MHLW Guidelines for the Use of Blood Products state, “In patients with chronic anemia due to MDS, administration of ESA preparations early before becoming transfusion-dependent may reduce transfusion volume” [9]. The question of whether transfusion can be avoided with ESA treatment before becoming TD needs to be studied further in real-world patients; unfortunately, the ESA-treated sample in this study was too limited to address the matter sufficiently. While this study examined the clinical and economic significance of reducing the requirement for RBC transfusions and/or maintaining NTD status, further research is needed from multiple angles to comprehensively evaluate this point. Recently, a phase 2 trial reported data on the efficacy and safety of luspatercept among Japanese NTD patients with LR-MDS [26], and luspatercept was approved in Japan for patients with anemia related to MDS regardless of transfusion dependency [11, 27]. However, since the number of patients enrolled in the phase 2 trial was limited (21 patients), further research is required to determine the efficacy of luspatercept in preventing transfusion dependence in NTD patients with LR-MDS in Japan.

Transfusion independence was associated with improved clinical outcomes; achievement of RBC-TI ≥ 8 weeks during weeks 1–24 among TD patients was associated with longer median overall survival (32.6 months) versus those who did not achieve RBC-TI (22.3 months), although the difference was not statistically significant. NTD patients who maintained NTD status also achieved longer median overall survival than those who did not, and median overall survival was not reached during weeks 1–24. In addition, the economic burden of transfusion dependence was pronounced; total direct adjusted medical costs were twice as high among TD patients when compared with NTD patients. Findings from this study also suggested that total medical costs may differ depending on whether transfusion independence was achieved or maintained among TD and NTD patients, respectively. Although the difference is less distinct among NTD patients, achieving and maintaining transfusion independence may have a positive impact not only on survival outcomes but also on total medical costs.

Limitations of this study include limitations inherent to the use of EHR data, particularly where the accuracy of the data included in the study relies on the accuracy of the records in the database [28]. Although the data presented include patients from the entire country of Japan and from geographically diverse regions, they do not represent all patients in Japan, and facility bias (where large, regional hospitals tend to include more severely ill patients) cannot be excluded. Furthermore, the study was performed during the COVID-19 pandemic period, which may have affected treatment patterns and associated study outcomes. The study was also limited by the fact that certain data (such as ring sideroblast status) were not available, and the information on IPSS/IPSS-R is based on the content in the medical records; therefore, its accuracy depends on the information recorded by medical professionals. Despite these limitations, the use of a large EHR database allows a comprehensive overview of the treatment patterns and outcomes for these patients in a community-based setting, which better reflects real-world patients than a clinical trial setting. However, due to the unavoidable limitations associated with this type of data, further examination with a larger number of cases is needed.

This study is the first to use an EHR database to examine real-world treatment patterns and clinical outcomes for patients with LR-MDS in Japan. Our findings suggest the clinical and economic importance of reducing the requirement for RBC transfusions and/or maintaining NTD status. Further research is required to understand the significance of early treatment and the impacts of new treatment options, including luspatercept, on patients with anemia related to LR-MDS in Japan.

## Supplementary Information

Below is the link to the electronic supplementary material.

Supplementary file1 (DOCX 236 KB)

## Data Availability

All data supporting the results of this study are included in the manuscript and its supplementary information. The Bristol Myers Squibb policy on data sharing may be found at https://www.bms.com/researchers-and-partners/independent-research/data-sharing-request-process.html.
