# Supplementary material for: Real-world treatment patterns and outcomes of patients with LR-MDS in Japan: an electronic health record database study
Source: Int J Hematol. 2025 Oct 2;123(2):196–207. doi: 10.1007/s12185-025-04077-y (PMC12913320; doi:10.1007/s12185-025-04077-y)
Supplement: Supplementary file 1 — Supplementary file1 (DOCX 236 KB) [file 12185_2025_4077_MOESM1_ESM.docx]

## Supplementary Information

**Supplementary Table 1.** Classification criteria used to confirm LR status (regardless of timing of risk registration)

| Criteria to confirm LR status, *n* (%) | Patients (*N* = 177) |
| --- | --- |
| Both IPSS and IPSS-R | 53 (29.9) |
| IPSS only | 60 (33.9) |
| IPSS-R only | 64 (36.2) |

*IPSS* International Prognostic Scoring System, *IPSS-R* Revised International Prognostic Scoring System, *LR* lower risk

**Supplementary Table 2.** Distribution of risk categories for IPSS and IPSS-R

| Risk category | Patients  *N* = 177 |
| --- | --- |
| IPSS, *n* (%) |  |
| Low | 40 (22.6) |
| Intermediate-1 | 72 (40.7) |
| Not evaluated per IPSS | 65 (36.7) |
| IPSS-R, *n* (%) |  |
| Very low | 7 (4.0) |
| Low | 43 (24.3) |
| Intermediate | 57 (32.2) |
| Not evaluated per IPSS-R | 70 (39.5) |

*IPSS* International Prognostic Scoring System, *IPSS-R* Revised International Prognostic Scoring System

**Supplementary Table 3.** Baseline clinical characteristics of patients who were treated with HMA and ESA as their first-line therapy

|  |  | HMAs |  |  | ESAs |  |
| --- | --- | --- | --- | --- | --- | --- |
|  | All patients | TD patients | NTD patients | All patients | TD patients | NTD patients |
| Hemoglobin, median (IQR), g/dL | ***n* = 57**  7.8 (6.6–9.9) | ***n* = 35**  6.9 (5.7–7.7) | ***n* = 22**  10.6 (9.6–11.9) | ***n* = 33**  8.0 (7.2–10.1) | ***n* = 17**  7.7 (6.7–8.0) | ***n* = 16**  10.1 (8.2–10.9) |
| Neutrophil count, median (IQR),  per μL | ***n* = 28**  1615.0 (800.0–5475.0) | ***n* = 14**  1302.5 (800.0–4400.0) | ***n* = 14**  3160.0  (800.0–5800.0) | ***n* = 15**  2100.0  (71.0–4500.0) | ***n* = 5**  1310.0  (330.0–5400.0) | ***n* = 10**  2135.0  (71.0–3900.0) |
| Platelet count, median (IQR),  × 10^4^/μL | ***n* = 57**  7.6 (2.8–12.4) | ***n* = 35**  5.5 (2.4–11.1) | ***n* = 22**  9.75 (3.8–12.9) | ***n* = 33**  13.9 (7.4–20.2) | ***n* = 17**  15.1 (5.6–21.3) | ***n* = 16**  13.15 (7.9–17.25) |

*ESA* erythropoiesis-stimulating agent, *HMA* hypomethylating agent, *IQR* interquartile range, *NTD* non-transfusion dependent, *TD* transfusion dependent

**Supplementary Table 4.** Outcomes among NTD patients who were not subject to “watchful waiting” but who started their first-line treatment without RBC transfusions

|  |  | ESA/ESA + Others/Others |  |
| --- | --- | --- | --- |
|  | Weeks 1–24  (*N* = 42) | Weeks 1–48  (*N* = 42) | Weeks 1–72  (*N* = 42) |
| Evaluable patients, *n/N* (%) | 35/42 (83.3) | 35/42 (83.3) | 32 (76.2) |
| No RBC transfusions, *n/N* (%) | 23/35 (65.7) | 17/35 (48.6) | 13/32 (40.6) |
| Started RBC transfusions, *n/N* (%) | 12/35 (34.3) | 18/35 (51.4) | 19/32 (59.4) |
| Non-evaluable patients, *n/N* (%) | 7 (16.7) | 7 (16.7) | 10 (23.8) |

*ESA* erythropoiesis-stimulating agent, *NTD* non-transfusion dependent, *RBC* red blood cell

**Supplementary Table 5.** Hemoglobin change by baseline transfusion status and line of therapy

|  | TD patients  (*n* = 79) | | NTD patients (*n* = 98) | |
| --- | --- | --- | --- | --- |
|  | Any line of therapy | First-line therapy | Any line of therapy | First-line therapy |
| **Weeks 1–24** |  |  |  |  |
| Hemoglobin change, g/dL |  |  |  |  |
| Mean (SD) | 0.37 (1.63) | 0.19 (1.51) | −0.75 (1.96) | −0.63 (1.90) |
| Median (range) | 0.07 (−3.0, 3.9) | 0.06 (−3.0, 3.6) | −0.25 (−11.0, 4.9) | −0.19 (−11.0, 4.9) |
| Hemoglobin increase ≥1.5 g/dL, *n* (%) | 18 (22.8) | 16 (20.3) | 5 (5.1) | 4 (4.1) |
| 95% CI | 14.1–33.6 | 12.0–30.8 | 1.7–11.5 | 1.1–10.1 |
| Hemoglobin increase <1.5 g/dL, *n* (%) | 61 (77.2) | 63 (79.7) | 93 (94.9) | 94 (95.9) |
| 95% CI | 66.4–85.9 | 69.2–88.0 | 88.5–98.3 | 89.9–98.9 |
| **Weeks 1–48** |  |  |  |  |
| Hemoglobin change, g/dL |  |  |  |  |
| Mean (SD) | 0.51 (1.63) | 0.21 (1.53) | −0.77 (1.97) | −0.63 (1.87) |
| Median (range) | 0.32 (−3.0, 5.1) | −0.02 (−3.0, 3.6) | −0.38 (−10.0, 4.9) | −0.16 (−10.0, 4.9) |
| Hemoglobin increase ≥1.5 g/dL, *n* (%) | 19 (24.1) | 16 (20.3) | 7 (7.1) | 6 (6.1) |
| 95% CI | 15.1–35.0 | 12.0–30.8 | 2.9–14.2 | 2.3–12.9 |
| Hemoglobin increase <1.5 g/dL, *n* (%) | 60 (75.9) | 63 (79.7) | 91 (92.9) | 92 (93.9) |
| 95% CI | 65.0–84.9 | 69.2–88.0 | 85.8–97.1 | 87.1–97.7 |

Adapted from Uno S, et al. Poster presentation at the 86th Annual Meeting of the Japanese Society of Hematology (JSH). Kyoto, Japan; October 11–13, 2024. Poster P-1-5-1 [1]

*CI* confidence interval, *NTD* non-transfusion dependent, *SD* standard deviation, *TD* transfusion dependent

**Supplementary Table 6.** Total direct adjusted medical costs in JPY and USD

|  | All patients | TD patients | | | | NTD patients | | | |
| --- | --- | --- | --- | --- | --- | --- | --- | --- | --- |
|  |  | Overall | Responder^a^ | Non-responder^a^ | Overall | | Responder^b^ | Non-responder^b^ |  |
| Total direct adjusted medical costs PPPM, average, (95% CI), JPY | 498,230.73 (498,206.14–498,255.31) | 728,799.41 (728,753.24–728,845.58) | 600,995.97 (600,946.65–601,045.28) | 1,062,169.56 (1,062,063.68–1,062,275.45) | 334,797.30 (334,770.95–334,823.65) | | 254,156.04 (254,127.95–254,184.14) | 496,721.50 (496,665.84–496,777.16) |  |
| Total direct adjusted medical costs PPPM, average, (95% CI), USD | 4612.99 (4610.63– 4615.36) | 6746.71 (6742.26– 6751.15) | 5559.61 (5554.87– 5564.35) | 9843.20 (9833.01– 9853.39) | 3100.56 (3098.02– 3103.09) | | 2354.32 (2351.61– 2357.02) | 4598.97 (4593.61– 4604.33) |  |

*CI* confidence interval, *JPY* Japanese yen, *NTD* non-transfusion dependent, *PPPM* per person per month, *TD* transfusion dependent, *USD* US dollars

^a^Achievement of RBC-TI ≥8 weeks during weeks 1–24. ^b^Achievement of RBC-TI ≥24 weeks during weeks 1–24

**Supplementary Fig. 1** Overall survival in patients with LR-MDS


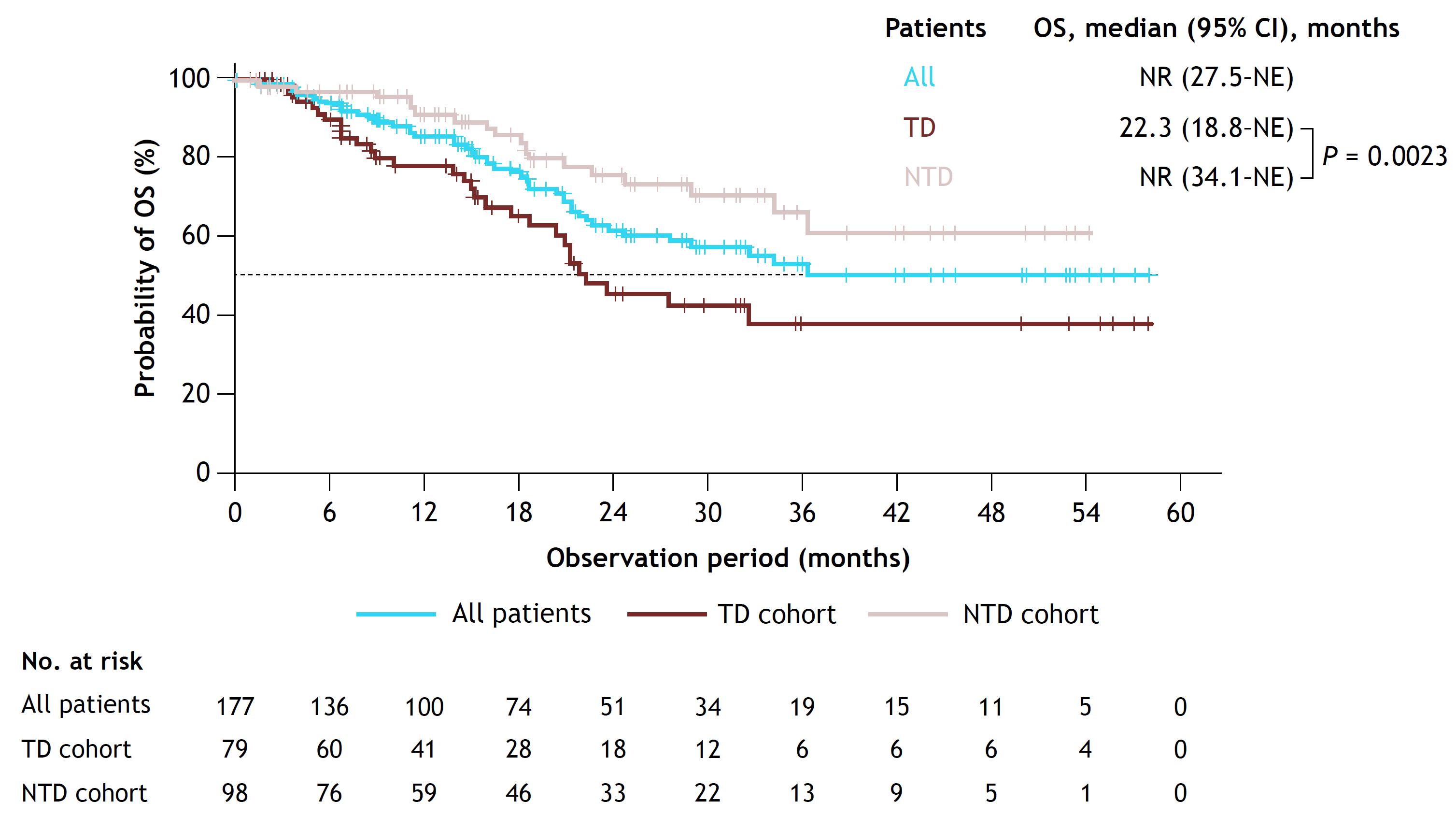


Adapted from Uno S, et al. Poster presentation at the 86th Annual Meeting of the Japanese Society of Hematology (JSH). Kyoto, Japan; October 11–13, 2024. Poster P-1-5-1 [1]

*CI* confidence interval, *LR-MDS* lower-risk myelodysplastic syndromes, *NE* not evaluable, *NR* not reached, *NTD* non-transfusion dependent, *OS* overall survival, *TD* transfusion dependent

## References

1. Uno S, et al. Poster presentation at the 86th Annual Meeting of the Japanese Society of Hematology (JSH). Kyoto, Japan; October 11–13, 2024. Poster P-1-5-1.
